# The Role of p38 MAPK in the Development of Diabetic Cardiomyopathy

**DOI:** 10.3390/ijms17071037

**Published:** 2016-06-30

**Authors:** Shudong Wang, Lijuan Ding, Honglei Ji, Zheng Xu, Quan Liu, Yang Zheng

**Affiliations:** 1 Cardiovascular Center, The First Hospital of Jilin University, Changchun 130021, China; wangshudong816@163.com (S.W.); wsd816@163.com (H.J.); wsxuzheng@163.com (Z.X.); 2 Department of Radiation Oncology, the First Hospital of Jilin University, Changchun 130021, China; juanjuan1693@163.com

**Keywords:** diabetic cardiomyopathy, p38 MAPK, cardiac dysfunction, microRNAs

## Abstract

Diabetic cardiomyopathy (DCM) is a major complication of diabetes that contributes to an increase in mortality. A number of mechanisms potentially explain the development of DCM including oxidative stress, inflammation and extracellular fibrosis. Mitogen-activated protein kinase (MAPK)-mediated signaling pathways are common among these pathogenic responses. Among the diverse array of kinases, extensive attention has been given to p38 MAPK due to its capacity for promoting or inhibiting the translation of target genes. Growing evidence has indicated that p38 MAPK is aberrantly expressed in the cardiovascular system, including the heart, under both experimental and clinical diabetic conditions and, furthermore, inhibition of p38 MAPK activation in transgenic animal model or with its pharmacologic inhibitor significantly prevents the development of DCM, implicating p38 MAPK as a novel diagnostic indicator and therapeutic target for DCM. This review summarizes our current knowledge base to provide an overview of the impact of p38 MAPK signaling in diabetes-induced cardiac remodeling and dysfunction.

## 1. Introduction

Diabetes mellitus remains a worldwide health problem, and is associated with a high rate of mortality, primarily as a consequence of cardiovascular complications. The diabetic complications of type 1 diabetes mellitus (T1DM) and type 2 diabetes mellitus (T2DM) have been extensively investigated. Diabetic cardiomyopathy (DCM) is a major diabetic complication. Diabetes-induced cardiovascular changes that lead to heart failure, which are independent of macro- or micro-vascular diseases, are believed to account for the high incidence of heart failure and mortality in diabetic patients [1,2]. DCM initially manifests as cardiac hypertrophy that can promulgate to the development of cardiac dysfunction, both in terms of diastolic and systolic function. A number of mechanisms have been proposed to explain how the diabetic environment stimulates the development of DCM, including mechanisms involving cardiac myocyte apoptosis [3,4], oxidative stress [2,5], inflammation [1] and remodeling [1]. For each of these pathogenic effects, mitogen-activated protein kinase (MAPK) signaling pathways are common denominator downstream targets. MAPKs, which include extracellular signal-regulated kinase 1/2 (ERK1/2), c-Jun N-terminal protein kinase (JNK), and p38 MAP kinase, regulate physiological and pathological processes [3]. MAPK pathways are upregulated with insulin resistance [6], cardiac hypertrophy [7,8] and heart failure [9,10,11]. Among the association links that have been established, the connection between p38 MAPK and DCM is the most extensively evaluated [8,12,13,14,15,16]. In this review, we summarize the current literature base to provide an overview of the roles of p38 MAPK in DCM.

## 2. Structure and Molecular Biology of p38 Mitogen-Activated Protein Kinase (MAPK)

Among the MAPKs, p38 MAPK is involved in a wide range of signaling pathways that stimulate a multitude of different biological functions [17]. The structure characteristics of p38 MAPK have recently been reviewed [17]. There are four isoforms in the p38 MAPK sub-family: p38α, p38β, p38γ and p38δ. The p38 MAPK isoforms are encoded by different genes and have different tissue-specific expression patterns. The p38*α* is ubiquitously expressed at significant levels in most cell types, while the others display more tissue-specificity. The p38β MAPK is highly expressed in the central nervous system and lung, p38γ MAPK is readily detected in skeletal muscles, and p38δ MAPK is enriched in endocrine glands. Among the p38 MAPK isoforms found in healthy heart, p38α is the major form; in which p38β shows low expression, and both p38γ and p38δ are minor components [18,19]. The diversity and specificity of cellular outcomes is achieved by functionally distinct p38 MAPK isoforms [20], with p38 MAPK regulating both cell survival [21] and physiological hypertrophy [22]. Accumulating evidence has indicated that p38α MAPK is essential for mammalian embryonic development, indicating a physiological role for this isoform [21,23]. Mice with a genetic deletion of the p38β MAPK survive and respond normally to inflammatory stimuli [24].

Akt activation is essential for hypertrophy responses to physiological stimuli [25]. Apoptosis signal-regulating kinase 1 (ASK1) null mice and cardiac specific p38α MAPK deficient mice developed an exacerbated form of physiologically cardiac hypertrophy through increased Akt activity in response to swimming, as an exercise stimuli [22]. In contrast, mice with cardiac specific overexpression of p38α MAPK display pathological hypertrophy in response to swimming [26]. Under inflammation or hypoxic conditions, the activation of p38α MAPK can suppress p38β MAPK, indicating the cross-talk among isoforms [27,28].

## 3. Effect of p38 MAPK Activation on Hearts of Diabetic Individuals

The p38 MAPK has been best described as having a key role in the pathophysiology of diabetes, particularly p38α MAPK [15,29]. The diversity and specificity of cellular outcome was achieved by functionally distinct p38 MAPK isoforms under different stress. There is evidence that p38 MAPK is activated during inflammation and oxidative stress, apoptosis, hypertrophy and energy metabolic abnormalities [4,30,31,32,33,34,35].

### 3.1. Inflammatory and Oxidative Stress Pathways

As a member of the MAPK family, p38 MAPK is specifically activated by phosphorylation in response to stress stimuli. The specific inhibition of p38α MAPK is necessary and sufficient to achieve anti-inflammatory efficacy, and p38β MAPK is not required for acute or chronic inflammatory responses [24]. Over the course of diabetes, several complications can occur, mostly due to hyperglycemia and elevated reactive oxygen species (ROS) production [36]. Complications include increased susceptibility to microbial infections and reduced capacity to clear the infection. In high glucose-containing medium or the hyperglycemic sera of T2DM, Wnt/β-catenin and p38 MAPK pathways are upregulated to impair dendritic cell differentiation and maturation [37]. Dendritic cell dysfunction caused by hyperglycemia could be responsible for increased susceptibility of diabetic individuals to infection. A p38 MAPK specific inhibitor, SB203580, partially rescues the impairment of dendritic cell differentiation and maturation induced by hyperglycemic sera, indicating the potentially important role of p38 MAPK in dendritic cell function [37]. ROS can activate p38 MAPK; and, in turn, p38 MAPK regulates the production of ROS to generate a feed-forward loop [38]; therefore, suppression of p38 MAPK can block ROS generation [39].

### 3.2. The Apoptotic Pathway

Cardiomyopathy is a late consequence of initial diabetes-induced early cardiac responses. One of the key early cardiac responses is cardiomyocyte apoptosis [3,40]. Hyperglycemia-induced ROS can activate MAPK to either stimulate or inhibit apoptosis in cardiomyocytes, depending on the isoform stimulated [35]. For example, p38α stimulation facilitates cardiomyocyte apoptosis [4]. In contrast, p38β stimulation is anti-apoptotic for cardiomyocytes [41]. Apoptosis mediated by p38 MAPK occurs through the upregulation of signal transducer and activator of transcription 1 (STAT1), C/EBP homologous protein (CHOP), focal adhesion kinase (FAK), similar to mothers against decapentaplegic homolog (SMAD), cytochrome c, nuclear factor (NF)-κB, phosphatase and tensin homolog (PTEN), and p53 pathways [17]. Functions of the other isoforms of p38 MAPK are not well-understood and need to be more completely addressed in future studies.

### 3.3. Pathological Hypertrophy

Hypertrophy occurs through both physiological and pathological mechanisms. Cardiac hypertrophy commonly occurs in response to pathological conditions such as diabetes, hypertension and myocardial infarction from coronary artery disease, which eventually results to cardiac fibrosis, remodeling and cardiac dysfunction. The sustained activation of p38 MAPK can lead to cardiac hypertrophy and dysfunction [42,43]. As a mediator, p38 MAPK phosphorylates and activates the GATA4 transcription factor to promote myocyte cell hypertrophy [44,45]. In addition, p38 MAPK regulates myocyte enhancer factor 2 (MEF2) transcriptional regulatory proteins to control cardiac differentiation during development [7,46].

### 3.4. Energy Metabolism Pathway

Fatty acid oxidation supplies greater than 50% of the energy needed for a normal adult heart to sustain contraction and metabolism, while glucose and lactate are the main energy sources for fetal hearts [47]. An unbalanced energy metabolism and myocardial lipid accumulation are early aberrant conditions in obese and insulin-resistant individuals [48]. Increased glucose uptake causes cardiac dysfunction, which is associated with the upregulation of p38 MAPK, as well as elevated ROS [42]. ROS upregulation in the diabetes can stimulate p38 MAPK and initiate mitochondrial dysfunction in cardiomyocytes [48]. Insulin receptor substrates 1 and 2 (IRS1 and IRS2) are activated to phosphorylate Akt, which regulates a variety of physiological functions involved with energy metabolism [49,50], myocardial growth [51] and survival [52].

The relationship between IRS1, IRS2, and p38α MAPK has been investigated in a recent study. Cardiac myocyte-specific IRS1 and IRS2 double null mice exhibited down-regulation of Akt phosphorylation, along with cardiac dysfunction [50]. Furthermore, IRS1 and IRS2 protein levels and Akt phosphorylation were reduced, whereas p38α MAPK phosphorylation was increased, in the hearts of high fat diet (HFD) and the leptin receptor deficient (db/db) mice, compared with control. These results revealed that p38α MAPK activation may be associated with reduction of IRS1 and IRS2 under diabetic or insulin resistant conditions. Next, the exact mechanisms whereby p38α MAPK regulates expression of IRS1 and IRS2 were determined by in vitro studies. IRS1 and IRS2 decreased with chronic insulin treatment along with increased p38α MAPK phosphorylation. Inhibition of p38α MAPK completely prevented the down-regulation of IRS1 and IRS2. In addition, overexpression of p38α MAPK downregulated IRS1 and IRS2 in a dose-dependent manner. The above results suggested that p38 activation was required for chronic insulin-induced IRS1 and IRS2 degradation and insulin resistance. [50]. In contrast, the overexpression of IRS1 or IRS2 attenuates p38α MAPK-dependent cardiac damage. Taking these results together, p38α MAPK mediates the effect of chronic insulin to promote insulin resistance by suppressing IRS1 and IRS2.

## 4. Protective Role of p38β MAPK in Diabetes

The cardioprotective role of p38β MAPK was universal in different animal models. Activation of the p38β MAPK attenuated doxorubicin-induced cardiotoxicity [53]. In an ischemic heart damage model, ischemia-related stress increased ROS generation, which in turn activates p38α MAPK to regulate p53 activity. Consequently, p53 activation inhibited p38β MAPK signaling cascade. This finding revealed a complex inter-relationship among p38 MAPK isoforms [28]. Similarly, under diabetic conditions, a few studies have demonstrated that p38β MAPK played an important role to protect against palmitate-induced endoplasmic reticulum stress and apoptosis in cardiac myocytes in vitro [41], and against palmitate-induced apoptotic effects in the heart of mice with fibroblast growth factor treatment [3].

In addition, the activation of p38β MAPK was also required for mediating the protective effect of stromal cell-derived factor-1β (SDF-1β) on cardiac myocytes exposed to lipotoxicity in vitro and diabetes in vivo [41]. In an in vitro study, inhibition p38 MAPK by SB203580 did not affect palmitate-induced cell death but completely abolished the protective effect of SDF-1β on palmitate-induced cell death. Since the SB203580 unselectively blocks the α and β of p38 MAPK, p38β MAPK siRNA was used to define the specific role of p38β MAPK in the protection of SDF-1β from palmitate-induced cell death since the protective effect of SDF-1β on palmitate-induced cell death was completely abolished by p38β MAPK siRNA. In summary, p38 MAPK can protect from and mediate various diabetes-induced pathological changes leading to DCM, depending on β isoform is activated.

## 5. The Inhibition of p38 MAPK Is Beneficial for Diabetic Complications

The inactivation or inhibition of p38 MAPK restores cardiac function in diabetes [15,29]. Atorvastatin, as a lipophilic statin, exerts beneficial effects in the prevention of cardiovascular disease and improves outcomes in patients with diabetes or obesity. In DCM, atorvastatin improves cardiac function by reducing inflammation and suppressing the activation of p38 MAPK [54]. Similar results were obtained in the kidneys of diabetic rats [55]. Atorvastatin treatment downregulated osteopontin (OPN) expression and improved kidney function along with suppressed phosphorylation of p38 MAPK. In an in vitro study, Madin-Darby canine kidney epithelial cells were pretreated with the pharmacological inhibitor of p38 MAPK (SB203580) or the pharmacological activator of p38 MAPK (phorbol 12-myristate 13-acetate, PMA). SB203580 inhibited glucose-induced p38 MAPK phosphorylation and correspondingly repressed OPN expression, while PMA increased the phosphorylation of p38 and the expression of OPN [55]. Gallic acid (GA, 3,4,5-trihydroxybenzoic acid) prevented the development of diabetic nephropathy by inhibiting p38 MAPK activation in high fat diet/STZ induced T2DM rats and cultured renal proximal tubular epithelial cells [56].

Insulin therapy is a primary method to treat diabetes, which can further prevent diabetic cardiac damage [57]. Early intensive insulin treatment (at the initial phase) and the attainment of good glycemic control reduce renal molecular pathways associated with epigenetic metabolic memory with the decline of p38 MAPK [58]. The above research displayed that p38 MAPK was involved in DCM and diabetic nephropathy, and suppression or downregulation of p38 MAPK improves cardiac function and kidney function. However, the protective effects are direct or indirect need to be further addressed.

### 5.1. Specific Inhibition of p38 MAPK with Inhibitors

The inhibition of p38 MAPK has been achieved using specific inhibitors, and genetic deletion, as shown in Table 1. Different inhibitors of p38 MAPK that target each of the four isoforms or inhibitors targeting a combination of these isoforms are available. The use of these inhibitors results in diverse effects. SB 203580 as an inhibitor of p38 MAPK does not distinguish between p38α and p38β and also reacts with other cellular targets including Akt and c-Raf [59]. SB 202190 and PD169316 blocks p38α and p38β, respectively. BIRB 0796 can suppress all four isoforms [59]. In streptozotocin (STZ)-induced diabetic cardiomyopathy, the inhibition of p38 MAPK with SB 203580 (1 mg/kg of body weight daily, beginning at the onset of diabetes and continued for eight weeks), improved cardiac function, which is associated with reduced cardiac inflammation characterized by reduced myocardial tissue necrosis factor α (TNF-α), interleukin-1β (IL-1β), and interleukin-6 (IL-6) levels [15]. In addition, the cardiac transforming growth factor-β (TGF-β) was decreased by the SB203580. These results revealed that inhibition of p38 MAPK by its specific inhibitor can prevents DCM in T1DM animal model.

In human adult ventricular cardiomyocytes (AC16 cells) exposed to high concentrations of palmitate to mimic diabetic lipotoxicity in the heart [4], palmitate induced the dose-dependent activation of p38 MAPK, with total p38MAPK levels remaining unchanged. The inhibition of p38MAPK with PD169316, a nonspecific inhibitor of p38 MAPK α and β, beginning at two hours prior to exposure to palmitate and continued for 16 h, reduced apoptosis induced by palmitate. The suppression of phosphorylated p38α MAPK with a specific siRNA against p38α MAPK attenuated cardiomyocyte apoptosis [4]. In a similar experimental condition, the inhibition of p38β MAPK with its specific siRNA abolished protective effect of stromal cell-derived factor-1 (SDF-1) on palmitate-induced apoptosis [41]. In addition, SB 202190 significantly decreases high glucose-induced inflammation [62].

In term of p38 MAPK inhibitor BIRB 0796, there was only one study showing that BIRB 0796 (1 µM, beginning at one hour prior to exposure to IL-1α) protected against excessive extracellular matrix protein accumulation in the myocardium during post myocardial-infarction remodeling [63]. However, there is no study with it directly to investigate the role of p38 MAPK on DCM yet; therefore, whether BIRB can be used to prevent against DCM remains further investigated.

### 5.2. Suppression of p38α MAPK in Dominant-Negative Mutant of Transgenic Model

Since the p38 MAPK inhibitor used in above studies does not distinguish between p38α and p38β, which isoform of p38 MAPK plays the role remains unclear. The specific isoforms of p38α or p38β transgenic models were used to investigate the role of p38α or p38β MAPK. The loss of p38α MAPK caused embryonic death, therefore, p38α MAPK knockout mice were not available to clarify the role of p38 MAPK in DCM. Accordingly, p38 MAPK transgenic mice with cardiac-specific overexpression of a dominant-negative mutant of p38α MAPK (TG DN p38α MAPK) has been used to investigate the role of p38α MAPK in a STZ-induced DCM [29]. In the diabetic mouse model, the expression of molecular markers of cardiomyocyte hypertrophy (ANP) and fibrosis (TGF-β and collagen III), as well as apoptotic cardiomyocytes, were elevated. These changes were markedly attenuated in transgenic mice. Cardiac dysfunction (reduced fractional shortening) induced by diabetes in WT mice was restored by TG DN p38α MAPK mice. These findings indicate that the p38α MAPK isoform plays an important role in cardiac damage associated with DCM in T1DM animal models.

T2DM is the most widespread metabolic disease in the world [64]. Further research is needed with p38 MAPK inhibitor or/and dominant-negative mutant of p38α MAPK transgenic mouse model of T2DM to fully understand the roles of p38 MAPK. Taken together, p38α MAPK is involved in the development of DCM, and p38β MAPK is required for the anti-apoptosis effects of inhibitors and gene deletion. Due to the existence of the four isoforms of p38 MAPK, using specific siRNAs against individual isoforms may be the best method to investigate the exact effect of each isoform for mechanistic evaluations.

## 6. Suppression of the Downstream of p38 MAPK

Clinical studies with p38 MAPK inhibitors have shown hepatotoxicity related to a p38 MAPK-mediated feed-back loop involving TGF-β activated kinase 1 (TAK1) and JNK activation [65,66]. The inhibition of the downstream of p38 MAPK may provide alternative targets including MAPKAPK-2 (MK2) and MAPKAPK-3 (MK3) to minimize or eliminate this complication. In most cells and tissues, MK3 expression is very low compared to MK2 expression. MK2 exacerbates inflammatory processes, and is necessary for the sustained activation of NF-κB, a central transcription factor in inflammation that has been reported to be involved in the development of insulin resistance [67]. MK2 is activated in the liver and heart. In diabetes, the suppression or deficiency of MK2 improves glucose tolerance and insulin sensitivity in obese mice [68,69,70,71]. The sarcoendoplasmic reticulum Ca^2+^-ATPase 2a (SERCA2a) is responsible for Ca^2+^ reuptake into the sarcoplasmic reticulum, and has been directly linked to contractility and alterations in excitation-contraction coupling [72]. Cardiac dysfunction in DCM has been shown to be associated with the downregulation of SERCA2a [73,74], similar to what is shown in ischemic cardiomyopathy [75,76] and stress-induced cardiomyopathy [77]. Protein kinase MK2, a p38MAPK downstream target, has been studied as a mediator in the development of DCM [30]. Five-week-old male control mice (MK2^+/+^) and MK2 whole-body null mice (MK2^−/−^) administered with STZ injections to induce diabetes have been observed for 15 weeks after the injections were initiated. The inhibition of MK2 improves insulin sensitivity and dyslipidemia. MK2^−/−^ mice exhibited improved cardiac function concomitant with normalized SERCA2a expression and phospholamban (PLB) phosphorylation. It is worth mentioning that the impaired metabolism of energy-providing substrates and myocardial lipid accumulation are early abnormalities in obese and insulin-resistant individuals. Free fatty acid (FFA) levels were elevated in positive control wild type diabetic mice, while these levels returned toward non-diabetic negative control mice levels in diabetic MK2^−/−^ mice. Furthermore, MK2^−/−^ mice revealed no changes in FFA metabolic rates, while diabetic positive control mice (MK2^+/+^) displayed enhanced exogenous FFA oxidation and fat esterification for storage. MK2 can directly regulate the expression of SERCA2a [72]. However, the deletion of MK2 prevents diabetes-induced cardiac dysfunction that may be related to improvements in systemic glucose tolerance and lipid profiles. In a short-term evaluation of diabetic nephropathy, it was found that MK2^−/−^ mice were not protected against renal hyperfiltration or elevations in glucose concentrations [78]. These different effects may be associated with a divergence in mechanisms across organs or across the duration of diabetes. Future studies using the cardiac specific deletion of MK2 are needed to investigate its beneficial effects in diabetic and obesity models that result directly from cardiomyocyte MK2 expression.

The inhibition of MK2/3 with the inhibitor, compound 28, improves glucose homeostasis in obese mice [70]. Compound 28 has additive beneficial metabolic effects with metformin in db/db mice, indicating the novel effect of MK2/3 inhibition. In light of the possible multiple beneficial effects of MK2/3 inhibition in the liver, effects on other organs need to be assessed. Of interest, the complete whole body deletion of MK2 aggravates the consequences of metabolic disorder in a high-fat diet induced obesity mouse model, due to enhancement of the pro-inflammatory polarization of adipose tissue macrophages and decreased expression of glucose transporter type (GLUT4) [71]. Compound 28 does not reduce GLUT4 levels, which may reveal differences between global genetic deletion and inhibition using a pharmacological inhibitor.

Taken together, MK2 or MK2/3 represents new potential therapeutic targets to T1DM-induced cardiac dysfunction and improve insulin sensitivity in T2DM. Differential effects between the genetic model and inhibitors needs to be reconciled as part of the attempt to translate these findings to clinic. Apart from MK2 and MK2/3, several other kinases activated by the p38 MAPK pathway are involved in the development of DCM, and these kinases include CREB, ATF1, NF-κB, and p53 [20]. The inhibition of these genes can protect against diabetic complications [79,80].

## 7. MicroRNAs Associated with p38 MAPK Activity in Diabetes

Emerging evidence supports roles for microRNA (miRNA) in heart disease, including the development of cardiac hypertrophy [81,82], myocardial ischemia [83], cardiac dysfunction [84] and metabolic stress [85]. miRNAs inhibit protein expression through binding to the 3’untranslated region (UTR) of target genes. The functions of these miRNAs can provide the central regulation of gene expression under conditions of stress (refer to reviews [86,87]). miRNAs are involved in the activation of p38 MAPK in a neonatal rat cardiomyocyte hypertrophy model induced by high glucose (HG) [88]. As shown in Table 2, p38 MAPK can be regulated as a target of multiple miRNAs [89]. Therefore, whether p38 MAPK related miRNAs are also involved in the pathological and intervention procedure of DCM is also interestingly discussed here.

In T1DM mice and in primary neonatal rat myocytes treated with HG, p38 MAPK upregulation occurs in association with the increased expression of hypertrophy markers ANP and brain natriuretic peptide (BNP) [88]. A miRNA microarray analysis revealed that miR-21, miR-208a and miR-705 are upregulated, while miR-29, miR-1, miR-373, miR-143, miR-20a, and miR-220b are downregulated in diabetic mice, compared to control mice. The upregulation of miR-373, which was achieved by transfecting cardiomyocytes with a miR-373 mimic, reduces hypertrophy and the expression of MEF2c, a transcription factor-associated myocardial hypertrophy. Inhibiting p38 MAPK using a specific inhibitor, SB203580, significantly reduces the expression of miR-373 and MEF2c, indictating that miR-373 is transcriptionally regulated by p38 MAPK.

miRNA-23b has recently been associated with the development of immune diseases [94] and in neovascularization during age-related macular degeneration [95] due to its key functions of cell cycle regulation, migration, apoptosis and differentiation [96]. miR-23b was downregulated in diabetic hearts and in cardiomyocytes following exposure to HG [90]. miR-23b overexpression attenuates HG-induced myocyte hypertrophy. In contrast, miR-23b reduction by treatment with a specific inhibitor induced cardiomyocyte hypertrophy. The suppression of p38 MAPK with SB203580 markedly increased miR-23b to reduce cardiomyocyte hypertrophy.

miR-143 expression is increased in DCM, leading to the inhibition of Akt signaling activity [91,97]. In the HL-1 cell line, insulin-mediated glucose uptake is lowered by 25% in cells expressing pre-miR-143 vs. control miRNA. The inhibition of miR-143 using the LNA inhibitor for miR-143 protected against the detrimental effects of DCM. The pharmacological p38 MAPK inhibitor SB203580 abolished the induction of miR-143, demonstrating that the induction of miR-143 is p38-dependent [91].

miR-24 is down-regulated in ischemic cardiomyopathy [98] and diabetes [99]. miR-24 suppresses cardiomyocyte apoptosis in a myocardial infarction model [98]. A novel mechanism for the development of DCM has been proposed, in which miR-24 is downregulated following the hyperglycemia-induced activation of c-Myc. Whether insulin resistance and hyperinsulinemia also mediate the expression or stability of miR-24 in diabetes remains unclear [100]. It has been reported that p38 MAPK is a direct target of miR-24 in humans [101] and in mouse models [92]. In Goto–Kakizaki rats, increased p38 MAPK levels were observed in an animal model of spontaneous T2DM. miR-24 was downregulated in muscles of Goto–Kakizaki rats, while the activation of the upstream of p38 MAK has been explored by testing relative luciferase activity. miR-24 replacement therapy is a promising avenue for diabetic patients. However, future studies in large animal models are warranted.

miR-21 prevents ischemia/reperfusion (I/R)- and H_2_O_2_-induced cardiomyocyte apoptosis by suppressing the Fas ligand and activating Akt [102]. Furthermore, miR-21 inhibition reduces p38 MAPK activation in A-498 cells [103]. It is worth mentioning that miR-21 increased significantly in a time-dependent manner in cardiac fibroblasts treated with high levels of glucose, and this increase is accompanied by an increase in p38 MAPK [93]. This implicates miR-21 as being involved in diabetes-induced cardiac fibrosis. The inhibition of miR-21 blocks the elevation of phosphorylated p38 MAPK to decrease fibrosis in the setting of DCM, demonstrating a direct role for miR-21.

In summary, a variety of miRNAs have been found to be involved in the p38 MAPK-mediated cardiac effects of DCM, where they act as either up-stream regulators or down-stream targets. The role of miRNAs in DCM is a burgeoning topic.

## 8. Conclusions

**Figure 1:**
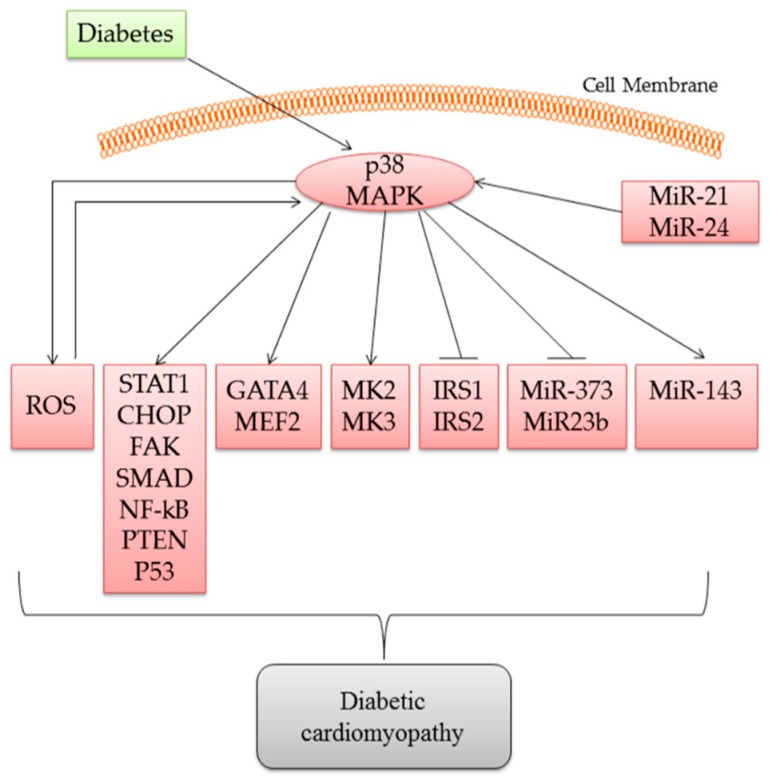
Activation of p38 Mitogen-activated protein kinase (MAPK) regulates a variety of pathological processes through multiple different signaling pathways.

There is increasing evidence that p38 MAPK plays a significant role in diabetic cardiomyopathy, including the regulation of diabetic complications such as cardiac hypertrophy, fibrosis, and apoptosis (Figure 1). Further work is needed to understand the complete role of p38 MAPK in the pathogenesis of cardiac remodeling and dysfunction before this knowledge can be applied in translational research, in order to assess the therapeutic efficacy of using p38 MAPK inhibitors for targeting diabetic complications.

## Figures and Tables

**Table 1:** Inhibitors of p38 MAPK isoforms.

Inhibitors	Isoforms	Model	Response	References
SB203580	α, β	Multiple injections of STZ (50 mg/kg i.p. for five days) in C57/BL6 mice	Improved cardiac function	[15]
SB202190	α, β	Single injection of STZ (65 mg/kg i.p.) in rats	Prevented cardiomyocyte apoptosis	[60]
PD169316	α, β	Human adult ventricular cardiomyocytes treated with palmitate	Increased vasorelaxation	[4]
BIRB 0796	α, β, γ and, δ	Cardiac myofibroblasts treated with 10 ng/mL of IL-1α for six hours	Reduced inflammatory cytokine release	[61]

p38 MAPK, p38 mitogen-activated protein kinase; IL, interleukin; i.p., intraperitoneal injection; STZ, streptozocin.

**Table 2:** MicroRNAs involved in p38 MAPK activity.

MicroRNA	Location	Model	Response	References
miR-373	Downstream	Single injection of STZ (150 mg/kg i.p.) in C57/BL6 mice	Prevent cardiomyocyte hypertrophy	[88]
miR-23b	Downstream	LV of T2D patients and cardiomyocytes from rat high glucose-induced model	Prevent cardiomyocyte hypertrophy	[90]
miR-143	Downstream	Primary rat cardiomyocytes exposed to adipose tissue from T2D patients	Increase cardiomyocyte insulin resistance	[91]
miR-24	Upstream	T2D patients and Goto-Kakizaki (GK) rat	Prevent cardiomyocyte apoptosis	[92]
miR-21	Upstream	Rat cardiac fibroblasts with high glucose treatment (in vitro)	Prevent cardiac fibrosis	[93]

Location refers to being upstream or downstream of p38 MAPK; i.p., intraperitoneal injection; LV-left ventricle; STZ, streptozotocin; T2D, Type-2 diabetes.

## Abbreviations

ANP	Atrium natriuretic peptide
ASK1	Apoptosis signal-regulating kinase 1
BNP	Brain natriuretic peptide
CHOP	C/EBP homologous protein
DCM	Diabetic cardiomyopathy
ERK1/2	Extracellular signal-regulated kinase 1/2
FAK	Focal adhesion kinase
FFA	Free fatty acid
GLUT4	Glucose transporter type 4
HG	High glucose
IL-6	Interleukin-6
IRS1	Insulin receptor substrates 1
IRS2	Insulin receptor substrates 2
JNK	C-Jun N-terminal protein kinase
I.P	Intraperitoneal injection
MEF2	Myocyte enhancer factor 2
MAPK	Mitogen-activated protein kinase
MK2	MAPKAPK-2
MK3	MAPKAPK-3
NF-κB	Nuclear factor-κB
P38 MAPK	P38 MAP kinase
PTEN	Phosphatase and tensin homolog
ROS	Reactive oxygen species
SERCA2a	Sarcoendoplasmic reticulum Ca^2+^-ATPase 2a
SDF-1β	Stromal cell-derived factor-1β
SMAD	Similar to mothers against decapentaplegic homolog
STAT1	Signal transducer and activator of transcription 1
STZ	Streptozotocin
T1DM	Type 1 diabetes mellitus
T2DM	Type 2 diabetes mellitus
TNF-α	Tissue necrosis factor α
TGF-β	Transforming growth factor-β
